# Non-Newtonian blood rheology significantly alters hemodynamic predictions during cardiac looping: a computational study

**DOI:** 10.3389/fbioe.2026.1892144

**Published:** 2026-09-23

**Authors:** Matthew C. Watson, Erica C. Kemmerling, Lauren D. Black

**Affiliations:** 1 Department of Biomedical Engineering, Tufts University, Medford, MA, United States; 2 Department of Mechanical Engineering, Tufts University, Medford, MA, United States; 3 Cellular, Molecular and Developmental Biology Program, Graduate School of Biomedical Sciences, Tufts University School of Medicine, Boston, MA, United States

**Keywords:** cardiac looping, embryonic heart development, non-Newtonian blood flow, shear-thinning rheology, wall shear stress

## Abstract

**Introduction:**

Hemodynamic forces play a key role in early cardiac morphogenesis, yet many computational studies assume Newtonian blood behavior. Here, we evaluate the impact of non-Newtonian shear-thinning rheology on flow patterns, pressure distributions, and wall shear stress (WSS) during cardiac looping using idealized three-dimensional models of the embryonic heart tube.

**Methods:**

Five geometries representing progressive looping stages, from a linear tube to an S-shaped configuration with ventricular ballooning, were analyzed under pulsatile flow using both Newtonian and power-law viscosity models.

**Results and Discussion:**

Across all stages, Reynolds numbers (Re ≈ 1–7) and Womersley numbers (Wo ≈ 0.3) indicated laminar, quasi-steady flow consistent with embryonic conditions. Incorporating shear-thinning rheology produced substantial deviations from Newtonian predictions, with peak systolic WSS differing by up to ∼94% (range 64%–94% depending on stage) and pressure drops by up to ∼70% (range 48%–70% depending on stage). These effects were most pronounced in regions of increased curvature and geometric complexity. These findings demonstrate that non-Newtonian rheology can substantially alter predicted hemodynamic quantities during cardiac looping and should therefore be considered in future developmental hemodynamic models, particularly when wall shear stress and pressure magnitudes are used to infer mechanobiological responses.

## Introduction

The heart is the first functioning organ to form in embryonic vertebrates—its first contractions begin around day 21 of embryonic development in humans (Foo et al., 2020; Günthel et al., 2018; Bark et al., 2017; Männer et al., 2010). The heart begins as a linear tube, consisting of a single layer of endothelial cells surrounding the lumen and a layer of primitive cardiac extracellular matrix, called cardiac jelly, separating the endothelium from an outer layer of beating myocytes. Over several days, the linear heart tube undergoes a process called “cardiac looping” to transform into its mature, four-chambered shape. While genetic and biological cues also play a role in proper heart formation and function, it has long been speculated that hemodynamics modulate cell signaling and tissue function via physical cues such as wall shear stress. In many cases, altered blood flow has been correlated with pathological heart formation, resulting in defects such as hypoplastic left heart syndrome (Bark et al., 2017; McCormick and Tzima, 2016; Bove et al., 2003). While many studies have focused on hemodynamics in the developing ventricle and chambered hearts (Wiputra et al., 2016; La et al., 2016; Saber et al., 2003; King et al., 1994), fewer studies have examined blood flow in the primitive embryonic heart tube. An accurate understanding of hemodynamics throughout cardiac looping could lead to a more complete understanding of cardiac development and could help elucidate cardiac abnormalities and pathologies that occur during embryonic development.

There are many physical forces present during cardiac looping, and the direct mechanical methods that drive mechanical looping remain poorly understood (Taber et al., 2010). Blood flow begins around the third gestational week in humans while the heart is a linear tube without valves or chambers (Bark et al., 2017). Blood flow exerts two stresses—wall shear stress and hydrostatic pressure—on the early heart walls (McCormick and Tzima, 2016). Cardiac cells can sense and respond to these stresses, and deviations from normal physiological stresses can result in serious pathologies (Bove et al., 2003; Peshkovsky et al., 2011; Taber, 1998). Thus, an understanding of the magnitudes of these stresses and how they change throughout embryonic cardiac development could help elucidate the causes of pathologies and offer insight into corrective procedures.

It has been well established that blood flow is critical for healthy cardiac development; however, it is difficult for *in vivo* experiments to capture the fluid mechanics present during heart development in humans. Computational fluid dynamics (CFD) simulations have emerged as a promising tool evaluate cardiac flow features such as wall shear stresses and pressure distributions. In recent years, CFD has been a tool to investigate blood flow during congenital heart disease (Pennati et al., 2013; Feinstein et al., 2012), as well as for studying the underlying fluid mechanics present during cardiac development (Vedula et al., 2017). More recently, developmental cardiovascular modeling studies have combined image-based reconstruction, advanced imaging modalities, and CFD to quantify hemodynamic forces in embryonic and fetal hearts and to investigate the role of flow-mediated mechanical cues in both normal and pathological cardiac development. These studies have emphasized the importance of accurately characterizing wall shear stress, pressure distributions, and local flow environments while highlighting ongoing challenges associated with imaging resolution, moving boundaries, and model validation in developing cardiovascular systems (Giesbrec et al., 2025; Salman et al., 2021; Salman and Yalcin, 2021).

Most CFD models which investigate embryonic heart looping assume a Newtonian model of blood, due to the low hematocrit of embryonic blood. In adult vertebrates, hematocrits are usually greater than 40% (Thurston and Henderson, 2006), and non-Newtonian behavior is exacerbated due to increased interactions between deformable red blood cells. Embryonic blood has a hematocrit that ranges between 20% and 30% (Brooks et al., 1970), lower than adult blood, but still non-Newtonian in behavior. Previous models have shown that using a Newtonian model in a physiologically-motivated geometry can result in substantial differences in predicted flow fields relative to shear-thinning models (Cherry and Eaton, 2013). For example, in straight tubes, blunted velocity profiles and higher-pressure gradients are observed in shear-thinning simulations compared to Newtonian simulations. Additionally, shear thinning flows in curved tubes have weaker secondary flows and less axial vorticity than Newtonian flows (Cherry and Eaton, 2013). We believe that it is necessary to incorporate a non-Newtonian model of blood and pulsatile flow to better simulate the fluid dynamics present during embryonic heart looping.

In this study, we present a CFD model that interrogates differences in hemodynamics during different stages of embryonic heart tube looping, specifically investigating differences in wall shear stress and pressure. We present data on the wall shear stresses and pressure distributions in non-Newtonian and Newtonian models of blood flow during embryonic heart looping. To our knowledge, this study is the first to systematically isolate the influence of shear-thinning blood rheology across a progression of idealized cardiac looping geometries spanning linear, C-looped, and S-looped morphologies with ventricular ballooning. By maintaining identical geometry-specific flow conditions between Newtonian and non-Newtonian simulations, this approach enables direct quantification of the influence of rheological assumptions on predicted wall shear stress and pressure throughout cardiac looping.

## Methods

### Heart tube stage geometries

Most CFD models use optical techniques, such as magnetic resonance imaging, cardio echography, or line scan confocal microscopy, to generate models of embryonic ventricles and animal models to obtain similar geometries (Vedula et al., 2017). While these optical techniques make it possible to generate complex models from late-stage embryonic heart ventricles, to the authors’ knowledge, current imaging approaches remain limited in their ability to resolve the earliest human heart tube stages *in utero* with sufficient spatial and temporal resolution for subject-specific computational modeling (Goenezen et al., 2012). Thus, our model uses idealized geometries, representative of dimensions of the developing embryonic heart tube, to study the hemodynamics present within early stages of cardiac looping. Idealized geometries have been used before to study embryonic heart tube development. For example, Taber et al. modeled the transition from oscillatory blood flow to pulsatile blood flow within an idealized two dimensional linear heart tube model with symmetric cardiac cushions (Taber et al., 2007).

We used idealized geometries representing five stages of cardiac looping, starting from a linear tube, transitioning through a C-shaped tube, and finally forming an S-shaped tube (Figure 1; Table 1). The five geometries were selected to represent progressive morphological features associated with cardiac looping rather than exact anatomical reconstructions. Stage 1 represents a linear heart tube, Stages 2 and 3 represent progressively curved C-loop morphologies, and Stages 4 and 5 represent S-loop morphologies with increasing ventricular ballooning. The primary objective was to isolate the influence of curvature, elongation, and lumen expansion on predicted hemodynamics while minimizing confounding effects from subject-specific anatomical variation. This approach is particularly important for early human heart tube stages, for which current *in utero* imaging techniques lack sufficient spatial and temporal resolution. Maximum lumen diameters and lengths (Table 1) were chosen to be representative of reported dimensions of the developing human embryonic heart tube (Wiputra et al., 2016; Taber et al., 2007), with lumen diameters increasing across stages to capture ventricular ballooning during looping. The largest lumen diameter (1 mm) in the Stage 5 model is comparable to reported chamber dimensions in early human embryonic ventricles (Wiputra et al., 2016). Similar idealized approaches have been widely used to interrogate mechanistic drivers of embryonic cardiac development (Taber et al., 2007), enabling controlled evaluation of specific geometric and rheological effects that would be difficult to isolate in anatomically reconstructed models.

**FIGURE 1:**
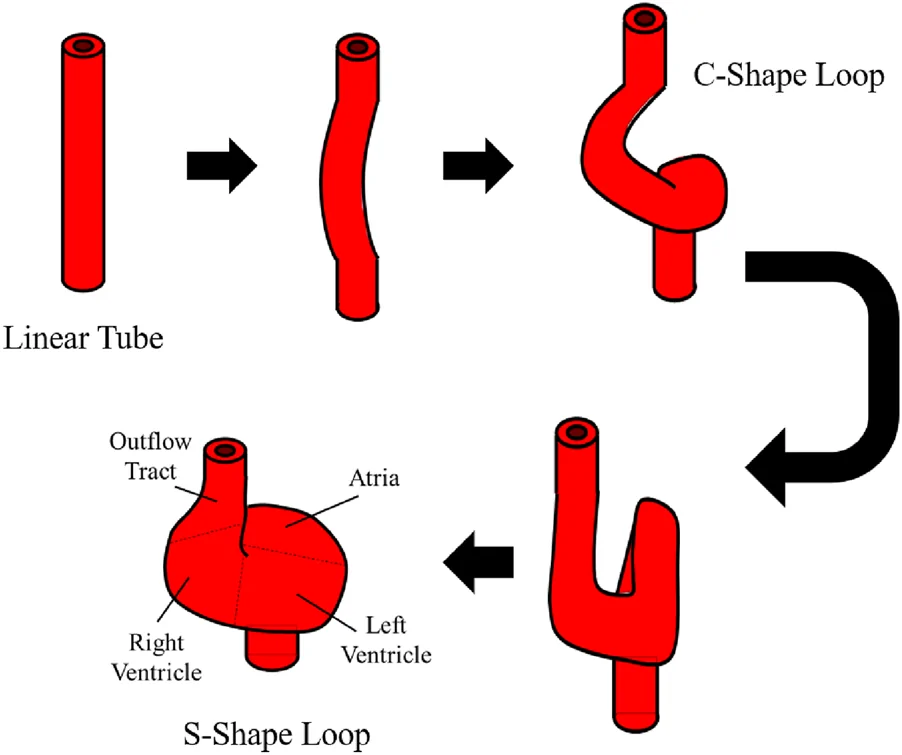
Schematic of the transition from the linear heart tube into, C-stage, and S-stage heart tube geometries. In addition to looping, the heart tube balloons and lengthens with the proliferating, stretching, and migrating cells.

**TABLE 1:** Summary of geometries and meshes.

Stage	Geometry	Max diameter (μm)	Centerline length (mm)	Number of Elements	Maximum element size (μm^3^)	Minimum element size (μm^3^)	Tortuosity
1	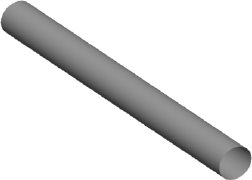	500	5	6.6E+04	3.2E+04	1.2E+03	1
2	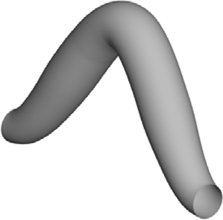	700	14	9.7E+04	8.1E+04	1.8E+03	1.56
3	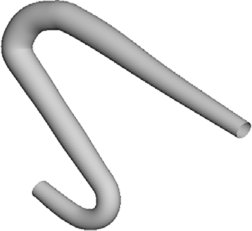	700	15	1.9E+05	2.5E+05	2.0E+03	3.15
4	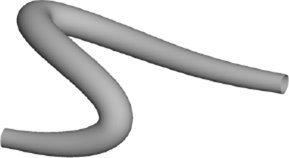	800	15	1.7E+05	2.1E+05	1.2E+03	2.42
5	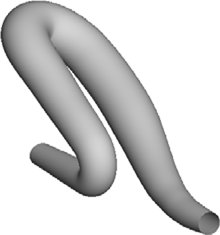	1000	15	1.8E+05	2.0E+05	1.9E+03	2.73

### Meshing

Table 1 summarizes the meshes and geometries for the five stages. Geometries were imported into ANSYS v19.2 workbench and meshes were generated for each stage using the ANSYS meshing software. Table 1 summarizes the number of elements for each geometry. Structured meshes featuring hexahedral elements with inflation near the lumen walls were used for all geometries. First, a growth layer five cells deep with 50 cells in the circumferential direction was applied to the face of the inlet boundary. The thinnest cell was at the edge and had a thickness of 3 µm. Inner cells had a growth rate of 1.2 in the radial direction. Mesh volumes were generated by sweeping the face grid along the outer radius of the geometries. A grid convergence study was performed to determine the minimum number of elements needed for key output variables, including peak wall shear stress and pressure drop, to vary by less than 1% between successive mesh refinements (See Supplementary Figure S1 for example, of refinement on Stage 2).

#### Blood material properties

Blood is a complex fluid that contains deformable cells. Despite this, many blood simulations have approximated blood as Newtonian. Rather, blood is non-Newtonian, and exhibits both shear-thinning and viscoelastic behavior due to interactions between red blood cells. Different methods have been used to model the non-Newtonian properties of blood. One common method is to model blood as a two-phase material featuring a Newtonian boundary layer without cells, and a core region rich with red blood cells (Medvedev and Fomin, 2011). Red blood cells naturally accumulate in the center of blood vessels, a phenomenon called the Fahraeus-Lindqvist effect. Two-phase models accurately capture this effect (Medvedev and Fomin, 2011); however, piecewise defined viscosity distributions are prone to discontinuous derivatives at the phase boundaries (Cherry and Eaton, 2013), and can lead to unstable solutions.

Another common technique for modeling blood viscosity is continuous viscosity functions (Cherry and Eaton, 2013; Sriram et al., 2012). These functions assign a viscosity to every point in the domain based on local shear rate, position, or time history. Continuous viscosity models have a relatively low computational cost, and can model both shear and viscoelasticity (Cherry and Eaton, 2013; McGinty et al., 2009; Moyers-Gonzalez et al., 2009). Several constitutive models have been proposed to describe the non-Newtonian behavior of blood, including Carreau-Yasuda, Casson, Cross, and Quemada formulations (Fuchs et al., 2023; Boyd and Buick, 2007). These models can provide improved representation of viscosity behavior at very low and very high shear rates. In the present study, a power-law model was selected because it provides a simple, continuous, and computationally efficient representation of shear-thinning behavior while accurately fitting experimentally measured viscosity data at embryonic hematocrit levels. Furthermore, the shear-rate range encountered in the present simulations primarily falls within the intermediate regime where power-law behavior provides a reasonable approximation of blood rheology. Because the primary objective of this work is to isolate the influence of shear-thinning behavior during cardiac looping, the power-law model provides an appropriate balance between physiological relevance, numerical robustness, and interpretability.

In our model, the viscosity of blood was modeled as a function of local shear rate. A power function (Equation 1) was used to fit data of shear rate dependence of blood viscosity for blood with a hematocrit of 28% from Brooks et al. (1970).

$$\mu =0.008781{\dot{\gamma }}^{-0.2307}$$ (1)

here, *µ* is the dynamic viscosity in *Pa·s* and $\dot{\gamma }$ is the local shear rate in 1/s. For shear rates less than 0.1 s^-1^, the viscosity was set to be a constant 0.0149 Pa·s. This lower shear-rate cutoff was implemented to prevent the nonphysical divergence in viscosity that can occur in power-law models as shear rate approaches zero. Because these very low shear-rate regions occupy a relatively small portion of the computational domain, the cutoff is not expected to substantially alter the primary comparisons between Newtonian and non-Newtonian simulations. A user defined function prescribed a viscosity value for each grid element within our geometries. Briefly, during each iteration, the local shear rate was calculated within each cell, and the viscosity of the fluid within each element was adjusted based on our power fit viscosity model.

Blood also has viscoelastic characteristics. The Weissenberg number (Wi) is a dimensionless number used to characterize viscoelastic flows (Equation 2) and can be interpreted as the ratio of elastic to viscous forces (Poole, 2012).

$$\text{Wi}=\dot{\gamma }\lambda$$ (2)

here, $\dot{\gamma }$ is defined as the shear rate and λ is the relaxation time (Poole, 2012). Relaxation time is defined as the amount of time a strained viscoelastic material takes to return to its initial equilibrium. Extensive rheology experiments conducted by Thurston *et al.* investigated Wi and relaxation times for blood in oscillatory flow in different flow geometries. For 22% hematocrit blood in a 0.10 cm diameter tube with an oscillatory frequency of 2 Hz, the maximum Wi was observed to be 0.1 at a shear rate of 12.7 s^-1^. Wi leveled off to 0.07 at shear rates greater than 100 s^-1^ (Thurston and Henderson, 2006). Based on the data from Thurston *et al.* (Thurston and Henderson, 2006) and and estimated shear rates within the developing embryonic heart tube, we expect Weissenberg numbers (Wi) to vary between 0 and approximately 0.1. This indicates that viscous forces dominate elastic effects under the flow conditions studied. Neglecting elastic stress terms within the governing equations therefore introduces a maximum expected error on the order of 10%. Notably, this error is substantially smaller than the differences observed between Newtonian and non Newtonian shear thinning models, which exceeded 40% for wall shear stress predictions at peak systolic flow. Consequently, a shear rate dependent viscosity model captures the dominant non Newtonian behavior of embryonic blood while maintaining numerical stability, computational efficiency, and interpretability of hemodynamic outcomes.

### Governing equations and boundary conditions

For incompressible flow, conservation of mass and momentum were solved using the continuity and Navier-Stokes (Equations 3, 4):

∇· u=0 (3)

$$\rho \left(\partial u/\partial t+u\;\cdot \nabla u\right)=-\nabla p+\nabla \cdot \;\tau$$ (4)

where u is velocity, ρ is density, p is pressure, and τ is the viscous stress tensor. For Newtonian simulations, viscosity was assumed constant, whereas non-Newtonian simulations employed the shear-rate-dependent viscosity relationship described in Equation 1. The governing equations were solved using ANSYS Fluent. For all cases, a no-slip boundary condition was used at the walls. The inlet velocity profile was specified as paraboloid-shaped with a transient flow rate at the inlet for both Newtonian and non-Newtonian models. A fully developed paraboloid inlet velocity profile was assumed to provide controlled and reproducible inflow conditions for systematic comparison across looping stages and viscosity models. Although embryonic inflow is generated by upstream peristaltic motion in a short, valveless heart tube, prior experimental and computational studies indicate that under the low to moderate Womersley numbers characteristic of early embryonic cardiac flow, velocity profiles rapidly approach quasi parabolic shapes over short axial distances (Goenezen et al., 2012; Taber et al., 2007; Lee et al., 2013; Courchaine and Rugonyi, 2018). Because all simulations applied identical inflow assumptions, relative comparisons of wall shear stress and pressure distributions between Newtonian and non-Newtonian cases remain robust, even if absolute inflow profile details vary *in vivo*. While the imposed inflow does not explicitly reproduce embryonic peristaltic pumping dynamics, the identical waveform and inlet assumptions applied across all simulations ensure that observed differences arise from rheological effects rather than inflow-condition variability. A mass flow rate vs. time waveform was obtained from previously published data by Taber et al. (Taber et al., 2007) and used as the flow rate waveform for our models (Figure 2A). Blood flow through the heart tube increases throughout cardiac looping and embryonic development (Figure 2B), and we accounted for this increase by scaling the mass flow rate waveform with published data of cardiac output data vs. gestational week (Hamill et al., 2011) (Figure 2C). Once the average mass flow rate of one pulse was determined for each stage, the average velocity vs. time was determined by dividing the flow rate by the cross-sectional area of the corresponding model. For all models, the derivative of pressure at the outlet was zero ( $\frac{\partial p}{\partial t}{|}_{outlet}=0$ and $\frac{\partial p}{\partial z}{|}_{outlet}=0$ ), and all pressures within the fluid domain are reported relative to the constant outlet pressure (Foo et al., 2020). A zero-gradient outlet condition was selected to avoid imposing uncertain downstream resistance values for the early embryonic circulation and to maintain consistency across all geometries and viscosity models. While more physiological outlet representations such as resistance or Windkessel models may alter absolute pressure values, the use of identical outlet conditions for all simulations ensures that observed differences arise primarily from rheological assumptions rather than differences in downstream loading. While cardiac output data were extrapolated to approximate early gestational stages, this approach introduces uncertainty in the absolute flow magnitudes predicted by the model. However, because identical scaling procedures were applied to both Newtonian and non-Newtonian simulations, the primary conclusions regarding relative differences in wall shear stress and pressure remain internally consistent. Consequently, the current study focuses on comparative rheological effects rather than exact prediction of embryonic cardiac output. Consequently, the present study should be interpreted primarily as a controlled comparison of rheological assumptions under identical inflow conditions rather than a fully physiological reconstruction of embryonic pumping dynamics.

**FIGURE 2:**
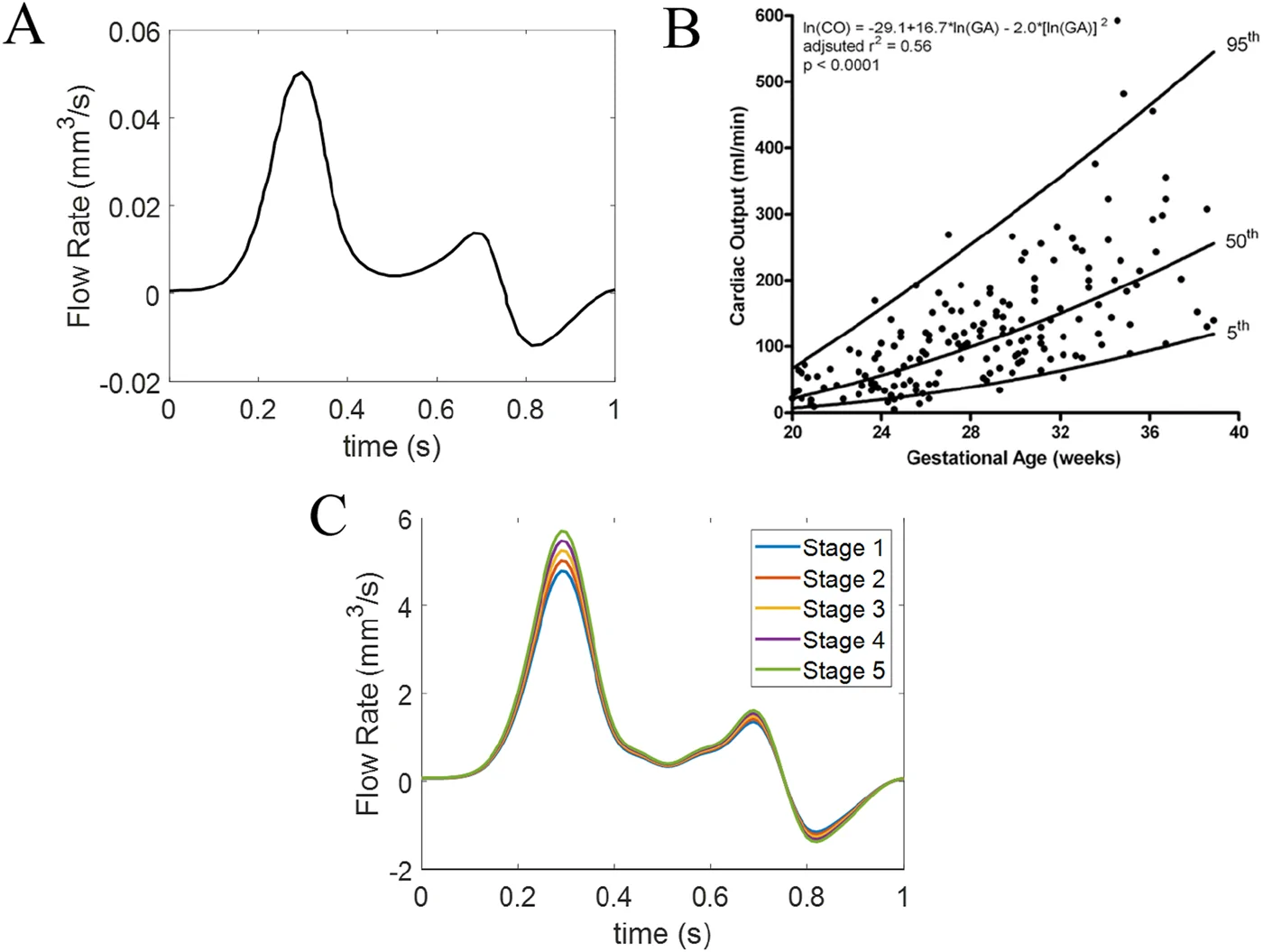
**(A)** Pulsatile flow rate computed from the outlet of a computational model of flow through a model of the chick embryonic linear heart tube (Taber 2007). **(B)** Data and fit of cardiac output vs. gestational age for healthy human patients between 20 and 40 weeks (Hamill et al., 2011). **(C)** Flow rate waveforms used by our models, matching the shape of the embryonic chick outflow waveform and the average cardiac output data extrapolated to 3 weeks.

#### Numerical methods

All simulations were performed in the commercial software Fluent using a second order upwind solver. For our models, we set the time step to be 0.002 s. All simulations were run for 500 time steps which equated to one cardiac cycle. Periodic behavior was not observed, and the final time step was used for analysis Scaled residuals are defined the imbalance between the left and right sides of the governing equations normalized by the largest error calculated during the first five iterations of each time step. For each time step, simulations were considered converged when scaled mass continuity residuals had converged to 1E-5% error.

#### Post-processing

Data was saved as ANSYS data and case files every ten time-steps for each case. Once simulations finished running, data was loaded into Tecplot 360 EX (version 2019 R1). Contour plots of wall-shear stress and hydrostatic pressure were generated for all geometries. Centerline coordinates of each geometry were extracted and used to create slices along inlet, outlet, and bends of each geometry. Contour plots of velocity and viscosity were generated for each slice.

## Results

### Validation of viscosity and velocity implementation

Contour plots of viscosity values were generated for both Newtonian and non-Newtonian cases of the mid cross-sectional plane of our Stage 1 geometry at peak systolic flow. Viscosity remained a constant 0.004 Pa·s for the Newtonian case, while the non-Newtonian case generated low viscosity values near the walls, and high viscosities near the center (Figures 3A,B). Vertical lines were drawn along the diameters of the mid cross-sectional planes for both cases. Velocity data were probed along the lines to generate velocity profiles. As expected, a parabolic shape is observed for the Newtonian model of flow Stage 1 geometry, while the non-Newtonian model featured a blunter and wider velocity profile (Figure 3C). This is expected for the non-Newtonian case: viscosities are low at high shear rates near the wall and high at low shear rates near the center of the vessel.

**FIGURE 3:**
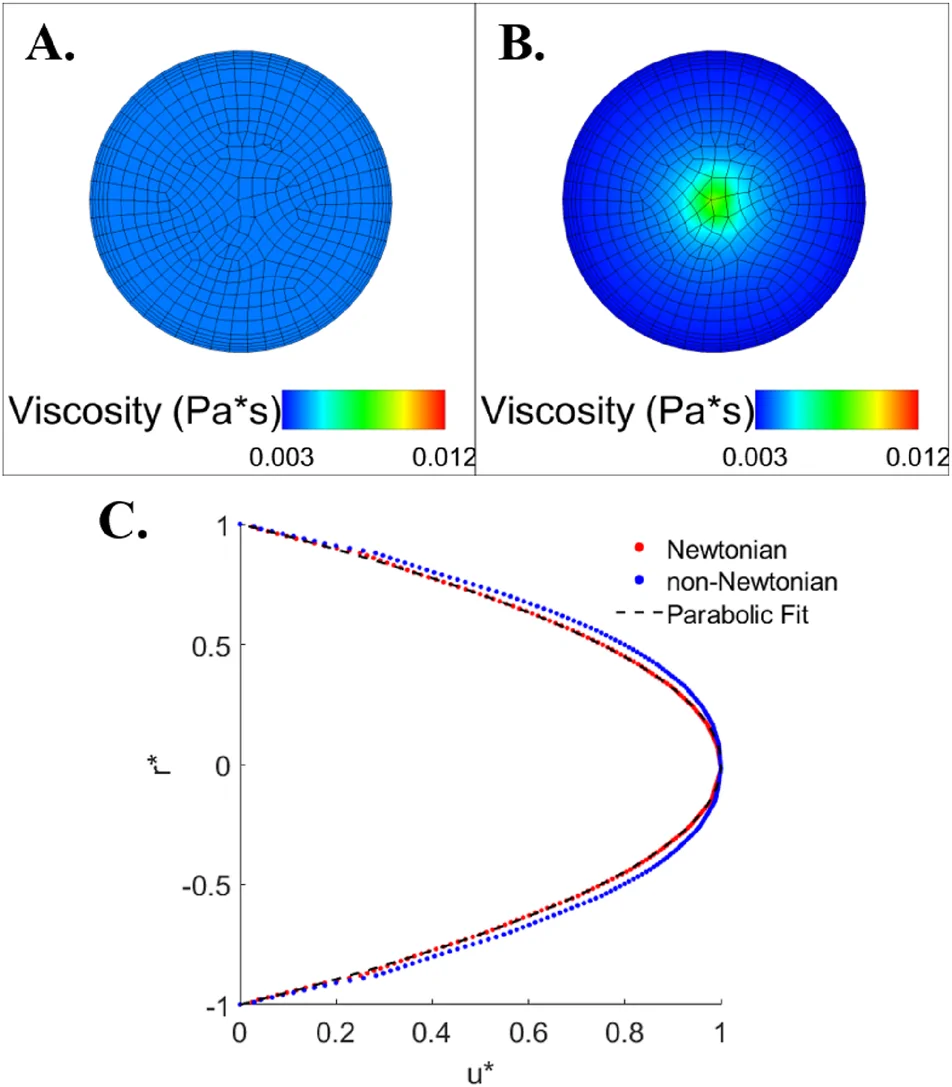
**(A)** Viscosity value of 0.004 Pa·s at peak diastolic flow through the Stage 1 Newtonian case. **(B)** Viscosity contours at peak diastolic flow through the Stage 1 non-Newtonian case. Contour plots were generated from data collect at the midway cross-section (*x* = 2.5 mm). **(C)** Velocity profiles for Newtonian and non-Newtonian Stage 1 cases at peak diastolic flow. r* is the normalized radial position defined *r*/*r*_*max*_ where *r*_*max*_ is the maximum radial postion. u* is the normalized axial velocity defined by *u/u*_*max*_ where *u*_*max*_ is the maximum axial velocity.

#### Hydrostatic pressure is overpredicted by Newtonian models

Contour plots of hydrostatic pressures were generated for both Newtonian and non-Newtonian cases at peak systolic flow and peak reverse diastolic flow (Figures 4, 5). In all models, we observed a monotonic pressure decrease from the inlet to the outlet. This is expected because the pressure gradient must balance viscous forces. Simulations predicted a decrease in pressure between stage 1 and stage 2 geometries possibly due to increased luminal diameters. Pressure drops increased between stage 2 and 3 geometries as expected with an increase in tube length. Finally, pressures dropped between stage 3 to stage 5 geometries where the tube length remained the same and lumen diameters increased. For all cases, Newtonian models of blood predicted higher pressure drops compared to non-Newtonian models. This is expected because lower viscosity values at high shear rates reduce flow resistance and pressures. Maximum pressure values for each stage and viscosity model at peak systolic flow are summarized in Table 2 together with the corresponding percent differences between Newtonian and non-Newtonian simulations.

**FIGURE 4:**
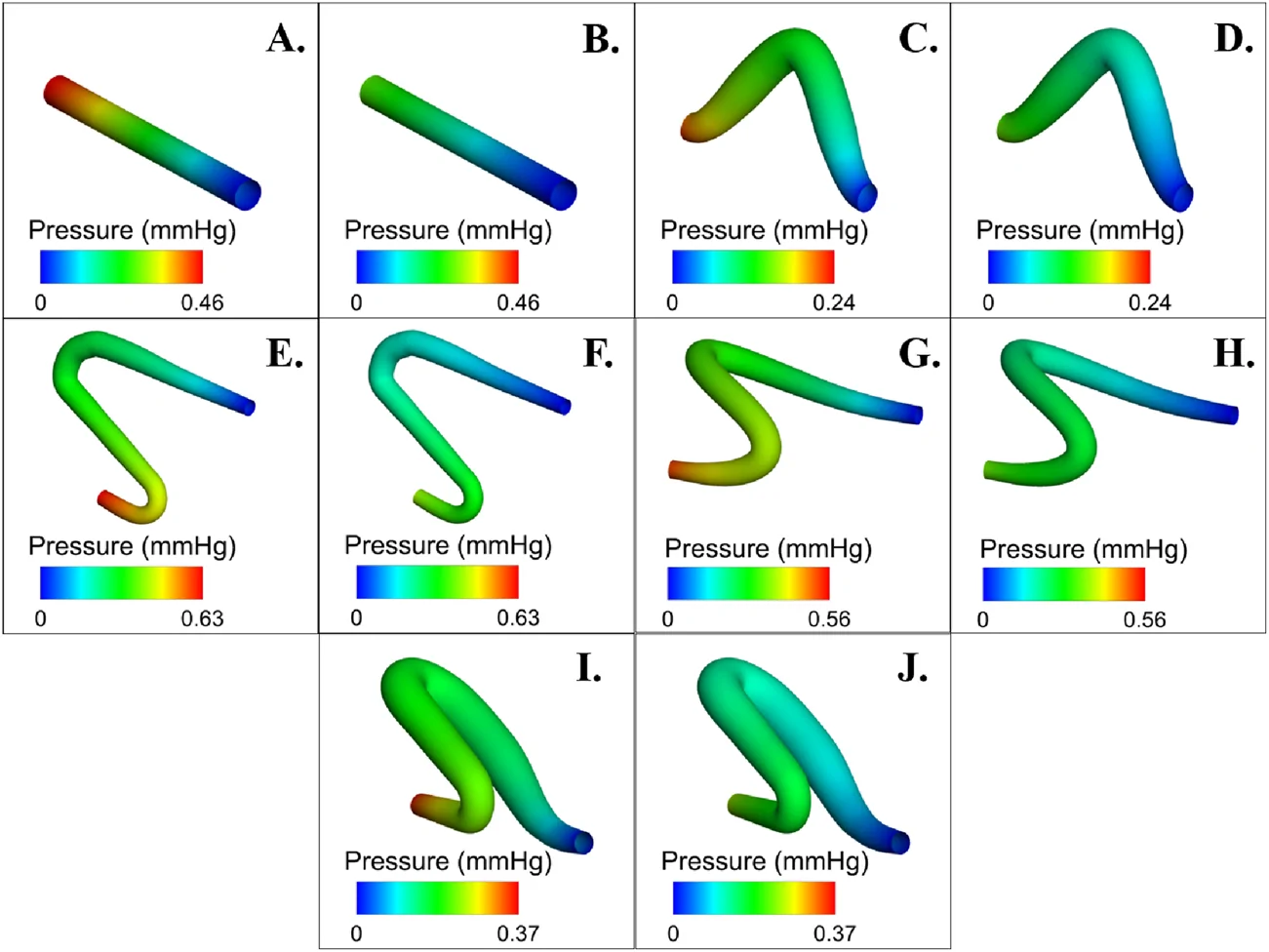
Peak systolic (t = 0.30 s) pressure values. Newtonian blood models: **(A)** Stage 1 **(C)** Stage 2 **(E)** Stage 3 **(G)** Stage 4 **(I)** Stage 5. Non-Newtonian blood models: **(B)** Stage 1 **(D)** Stage 2 **(F)** Stage 3 **(H)** Stage 4 **(J)** Stage 5. For all geometries, inlet diameter was held constant at 0.500 mm, while maximum lumen diameter varied by stage as reported in Table 1.

**FIGURE 5:**
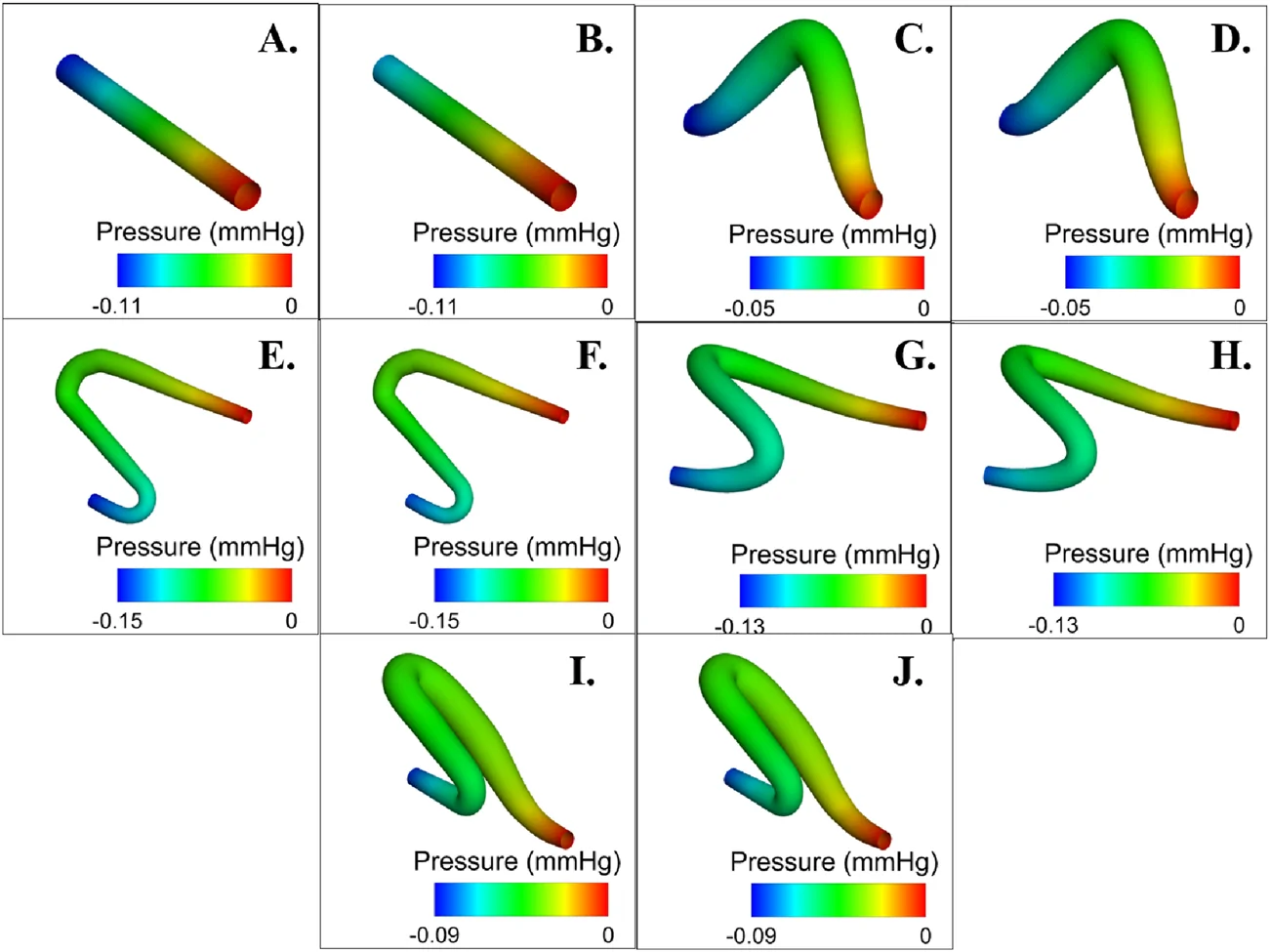
Pressure contours at peak reverse diastolic flow (t = 0.80 s). Newtonian blood models: **(A)** Stage 1 **(C)** Stage 2 **(E)** Stage 3 **(G)** Stage 4 **(I)** Stage 5. Non-Newtonian blood models: **(B)** Stage 1 **(D)** Stage 2 **(F)** Stage 3 **(H)** Stage 4 **(J)** Stage 5. For all geometries, inlet diameter was held constant at 0.500 mm, while maximum lumen diameter varied by stage as reported in Table 1.

**TABLE 2:** Maximum pressures at peak systolic flow.

Stage	Newtonian pressure (mmHg)	Non-Newtonian pressure (mmHg)	Percent difference
Peak systolic flow			
1	0.46	0.27	70%
2	0.24	0.15	60%
3	0.63	0.41	54%
4	0.56	0.36	56%
5	0.37	0.25	48%

Similar trends in pressure are predicted at peak reverse diastolic flow for all cases (Figure 5). Pressure values for diastolic flow are summarized in Table 3. Newtonian simulations predicted larger pressure values compared to non-Newtonian simulations; however, differences in pressure between Newtonian and non-Newtonian simulations were smaller at peak reverse diastolic flow than peak systolic flow. This is likely resultant from higher viscosity values at the lower diastolic shear rates.

**TABLE 3:** Maximum pressures at peak reverse diastolic flow.

Stage	Newtonian pressure (mmHg)	Non-Newtonian pressure (mmHg)	Percent difference
Reverse diastolic flow			
1	0.108	0.09	20%
2	0.055	0.051	8%
3	0.148	0.139	6%
4	0.131	0.121	8%
5	0.087	0.085	2%

### Wall shear stress comparison

Wall shear stress contour plots were generated for all geometries at peak systolic flow and peak reverse diastolic flow (Figures 6, 7). For all curved geometries during peak systolic flow (Stages 2–5), local maxima of wall shear stress occurred at the inlet, outlet, and innermost bends (Figures 6C–J). High shear stresses at the inlet and outlet are resultant from high shear rates where luminal diameters are smallest. Additionally, local maxima in wall shear stress at the innermost bends are from large velocity values shifting from the center to closer to wall. This resulted in larger shear rates near the innermost bend (Figure 8). For all stages, Newtonian simulations predicted higher shear stresses than non-Newtonian simulations for all geometries. This produced differences exceeding 40% between Newtonian and non-Newtonian simulations. This overprediction is likely explained by examining shear rates and viscosity values (Figures 3, 8). Velocity profiles for non-Newtonian cases had higher shear rates near the walls than velocity profiles for Newtonian cases. Higher shear rates resulted in decreased viscosity values near the wall for non-Newtonian cases and produced lower shear stresses than Newtonian cases. Maximum shear stresses predicted by simulations at peak systolic flow are summarized in Table 4 together with percent differences between rheological models.

**FIGURE 6:**
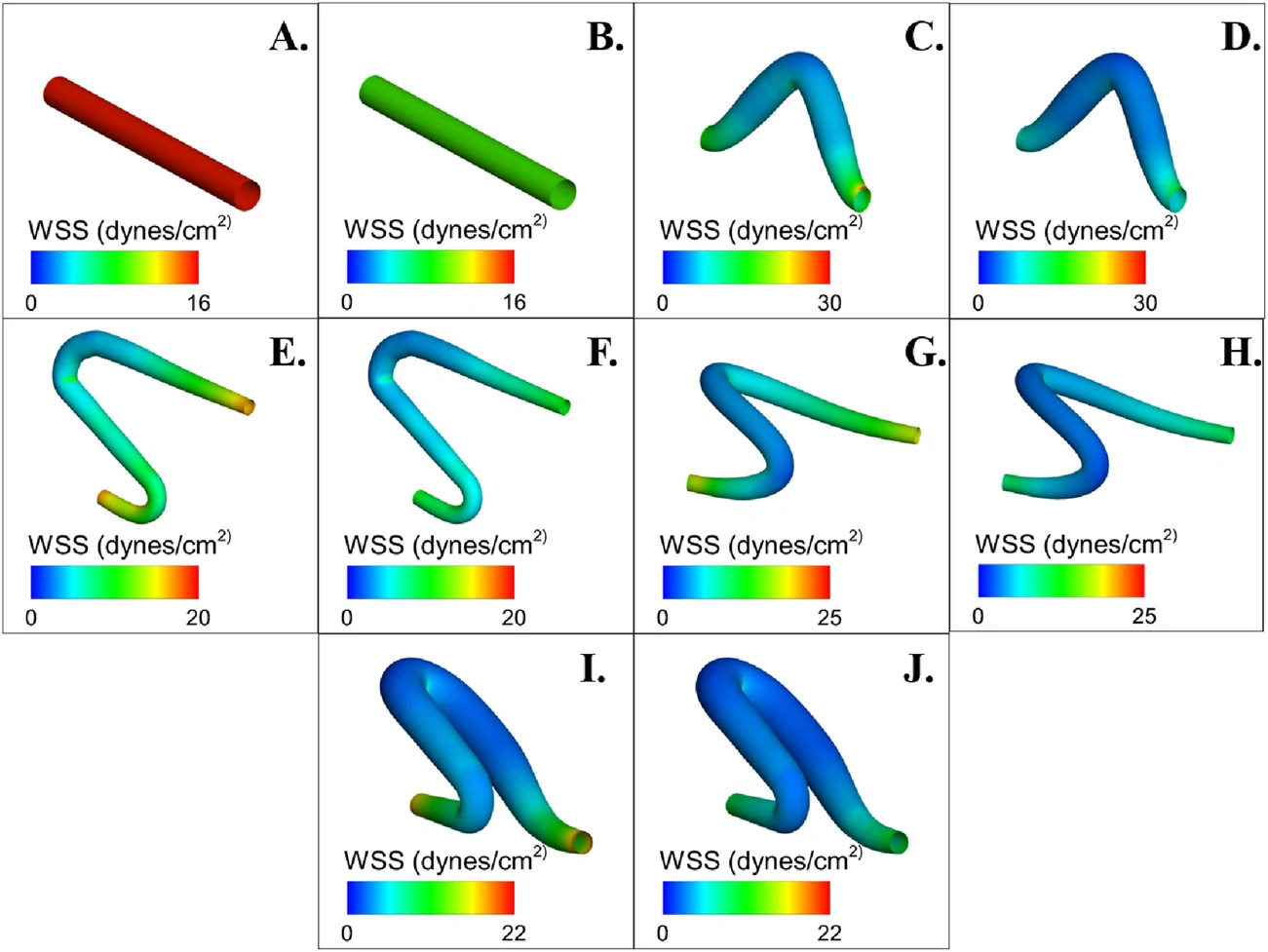
Wall shear stress contours at peak reverse diastolic flow (t = 0.80 s). Newtonian models: **(A)** Stage 1 **(C)** Stage 2 **(E)** Stage 3 **(G)** Stage 4 **(I)** Stage 5. Non-Newtonian models: **(B)** Stage 1 **(D)** Stage 2 **(F)** Stage 3 **(H)** Stage 4 **(J)** Stage 5. For all geometries, inlet diameter was held constant at 0.500 mm, while maximum lumen diameter varied by stage as reported in Table 1.

**FIGURE 7:**
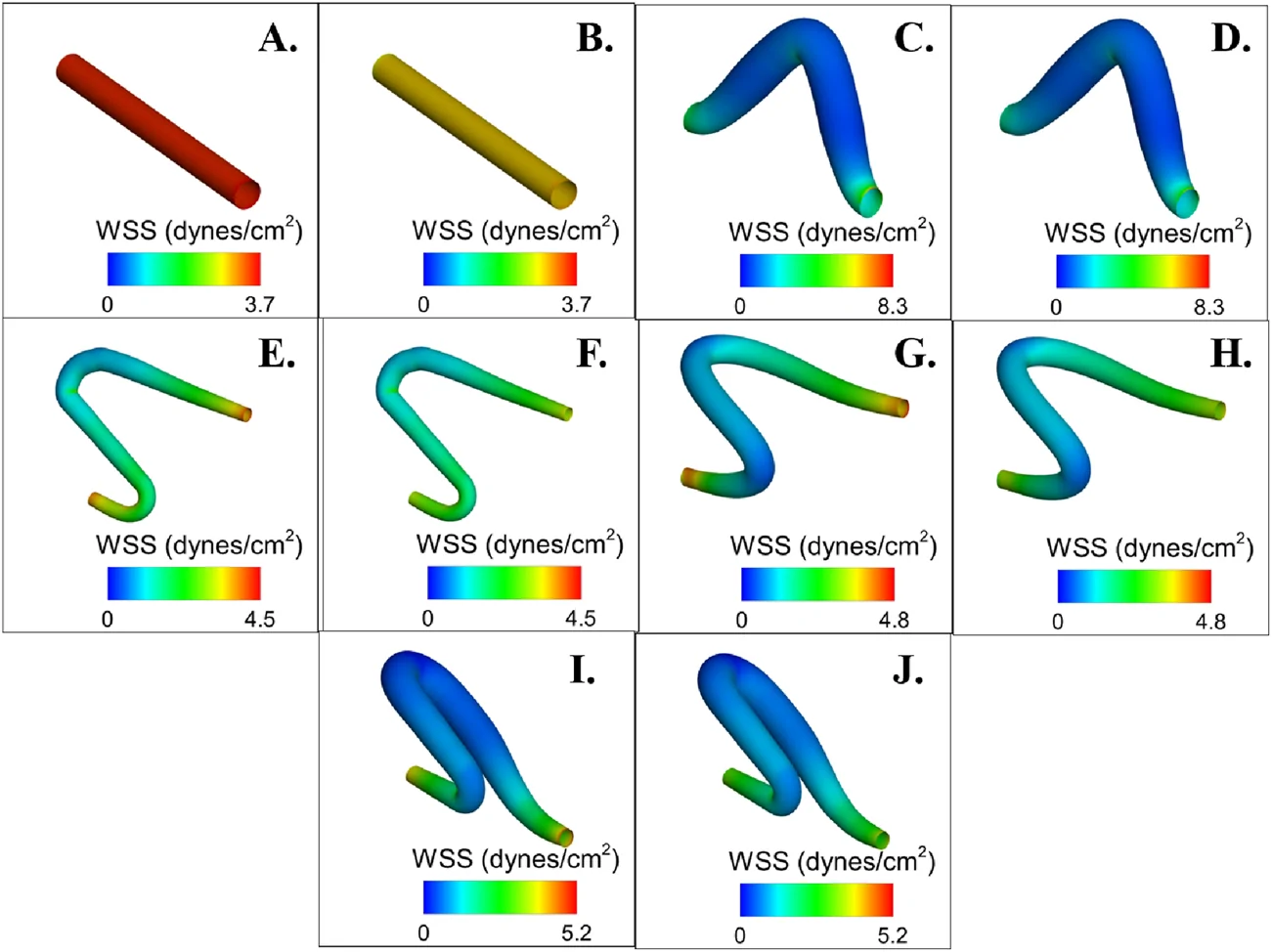
Wall shear stress contours at peak reverse diastolic flow (t = 0.80 s). Newtonian blood models: **(A)** Stage 1 **(C)** Stage 2 **(E)** Stage 3 **(G)** Stage 4 **(I)** Stage 5. Non-Newtonian blood models: **(B)** Stage 1 **(D)** Stage 2 **(F)** Stage 3 **(H)** Stage 4 **(J)** Stage 5. For all geometries, inlet diameter was held constant at 0.500 mm, while maximum lumen diameter varied by stage as reported in Table 1.

**FIGURE 8:**
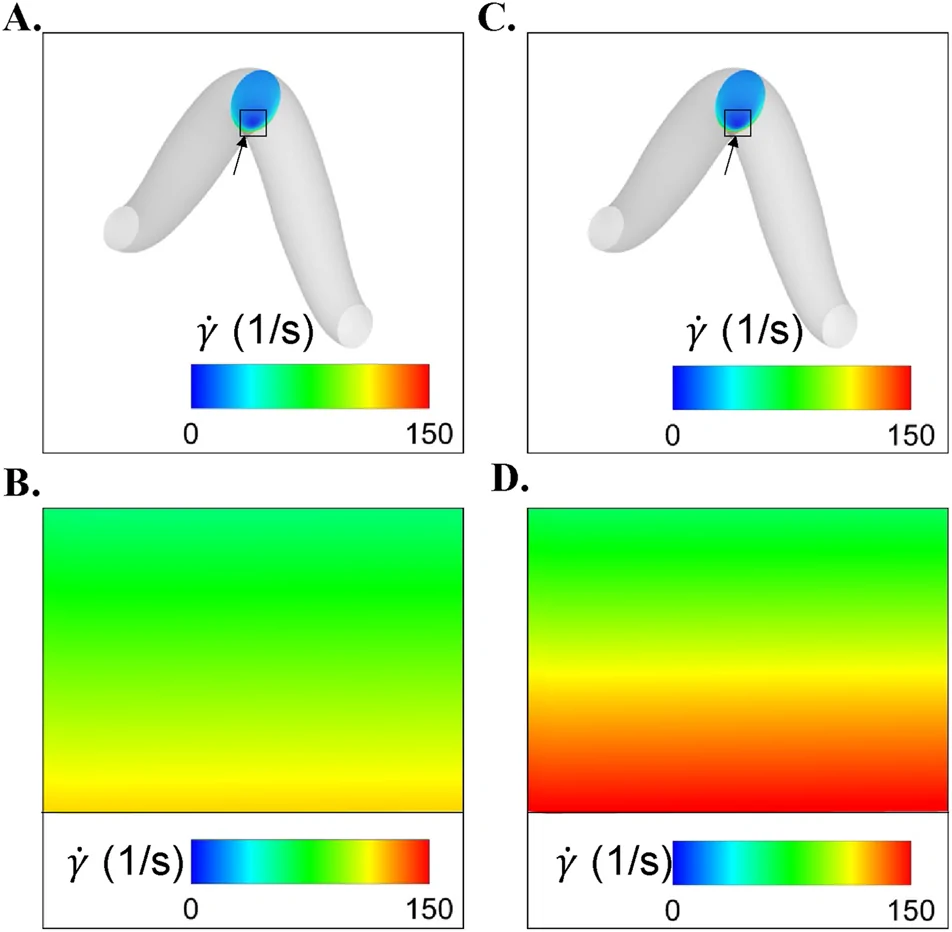
Contours of shear rate at a cross section through the bend in stage 2 geometries for Newtonian **(A,B)** and non-Newtonian **(C,D)** simulations at peak systolic flow. Black arrows in figures **(A,C)** denote the location zoomed sections of shear rate near the innermost walls in Newtonian **(B)** and non-Newtonian **(D)** cases.

**TABLE 4:** Maximum wall shear stress at peak systolic flow.

Stage	Newtonian wall shear stress (dynes/ cm^2^)	Non-Newtonian wall shear stress (dynes/ cm^2^)	Percent difference
Peak systolic flow			
1	15.9	9.1	75%
2	29.5	15.9	86%
3	19.4	11.8	64%
4	24.6	12.7	94%
5	21.5	12.1	78%

Our simulations predicted similar trends in shear stresses during peak reverse diastolic flow. For all stages, Newtonian simulations predicted larger shear stresses than non-Newtonian simulations, resulting in differences exceeding than 10%. This decrease between Newtonian and non-Newtonian flows is due to lower shear rates caused by lower velocities. For non-Newtonian cases at low shear rates, viscosity values increase also increasing shear stresses. This resulted in a smaller difference between Newtonian and non-Newtonian shear stresses. Maximum shear stresses predicted by simulations and peak diastolic flow are summarized in Table 5.

**TABLE 5:** Maximum wall shear stresses at peak reverse diastolic flow.

Stage	Newtonian wall shear stress (dynes/ cm^2^)	Non-Newtonian wall shear stress (dynes/ cm^2^)	Percent difference
Reverse diastolic flow			
1	3.7	3.1	19%
2	8.3	6.6	26%
3	4.5	3.9	15%
4	5.4	3.9	38%
5	5.2	4.2	24%

## Discussion

Mechanical stresses are associated with the onset of blood flow within the developing embryonic heart at around 3 weeks in humans (Taber et al., 2007; Krishnan et al., 2014). Cells residing within the embryonic heart are sensitive to these mechanical cues, and cardiac cells respond to maintain homeostasis and progress cardiac morphogenesis (Courchaine and Rugonyi, 2018; Watson et al., 2020; Chistiakov et al., 2017; Tarbell and Ebong, 2008; Davies, 1995; Haack and Abdelilah-Seyfried, 2016; Jacot et al., 2010). Determining physiological values of these mechanical stresses are necessary to understand cellular behavior during embryonic heart development (Taber et al., 2010; Taber, 1998; Taber et al., 2007; Lin and Taber, 1994); however, pathological values can lead to improper diagnostics, treatments, and understanding of congenital malformations that arise during heart development (Stekelenburg-de Vos et al., 2012). We present a computational model for pulsatile blood-flow through embryonic cardiac looping, accounting for the shear-thinning viscosity of blood. For all cases, we assumed a fully developed paraboloid velocity profile at the inlet.

We compared results to cases where blood was treated as a Newtonian fluid with a constant viscosity. Predicted wall shear stresses and pressure differences remained within the same order of magnitude (1–15 Pa) as values reported in previous experimental and computational studies of embryonic zebrafish, chick, and fetal cardiovascular development (Taber, 1998; Taber et al., 2007; Peterson et al., 2012; Jamison et al., 2013). Although direct validation against subject-specific human embryonic flow data is not presently feasible, agreement with published developmental hemodynamic ranges supports the physiological plausibility of the simulated flow fields. Our simulations predicted local maxima in wall shear stresses at bends in the looping geometries, like where primitive cardiac cushions develop (Taber et al., 2007), and evenly distributed wall shear stresses predicted by non-Newtonian models may alter the predicted mechanical cues present in regions associated with ventricular trabeculation; however, the current simulations do not directly evaluate downstream biological responses (Lee et al., 2016). Additionally, our simulations discovered that Newtonian simulations using a viscosity of 0.004 Pa s overpredicted pressures and wall-shear stresses when compared to non-Newtonian cases.

The present models assume rigid, stationary walls and therefore isolate fluid borne stresses acting on the endocardium during cardiac looping. *In vivo*, the embryonic heart tube is compliant and undergoes cyclic deformation; however, prior computational and experimental studies suggest that while wall motion modulates the magnitude of wall shear stress and pressure, the spatial localization of shear stress maxima—particularly at bends, inlets, and outlets—is preserved (Taber et al., 2007; Courchaine and Rugonyi, 2018). By focusing on relative differences between Newtonian and non-Newtonian rheology under identical geometric and flow conditions, our approach provides mechanistic insight into how blood rheology alone biases predicted hemodynamic loads during early cardiac morphogenesis.

Our simulations predicted wall shear stresses similar to stresses observed experimentally in zebrafish and chicken embryo models of cardiac morphogenesis (Peterson et al., 2012; Wang et al., 2006), and both simulations predicted peak systolic stresses at bends in the tube close to shear stresses observed during valve development in the embryonic S-staged zebrafish heart (Wang et al., 2006). Local maxima in shear stresses occurred at bend locations, where curvature is increased, or small luminal diameters. These locations are close to the locations of endocardial cushions, where valve development occurs later in heart morphogenesis (Bark et al., 2017; Jamison et al., 2013). Additionally, local maxima in wall shear stress at the inlet and outlet provide insight into the development of atria and the outflow tract. For example, work demonstrated that arteries can adjust their lumen diameters to maintain shear stresses near a set optimal point (Taber, 1998). Understanding what the optimal shear stress load is for normal outflow tract development could help identify conditions yielding outflow tract pathologies.

Healthy development of embryonic cardiac features are highly dependent on shear stress, and other studies have shown that cellular response to shear stress have been associated with ventricular trabeculation (Peshkovsky et al., 2011; Vedula et al., 2017), cardiac cushion formation (Taber et al., 2007), and valve differentiation (Forouhar et al., 2006; Hove et al., 2003). Gene-expression patterns associated with wall shear stress have been linked to both physiological and pathological heart formation. For example, overexpression of neuregulin-1 leads to over proliferative cardiomyocytes and can lead to hyper-trabeculation and CHDs (Parodi and Kuhn, 2014). Several other signaling pathways, such as *NOTCH, Nkx-2.5,* and *ENT1* linked to distributions and magnitudes of wall shear stress (Chen et al., 2004; Bruneau, 2008; Bruneau, 2013). To this extent, non-Newtonian simulations predicted more evenly distributed wall shear stress distributions than Newtonian simulations, which could affect ventricular trabeculation. Inaccurate reported wall shear stresses hamper our understanding of physiological and pathological embryonic cardiac development. By experimentally examining genetic expression under Newtonian and non-Newtonian shear stress conditions, scientists may be able to generate testable hypotheses regarding the relationship between flow-mediated mechanical cues and developmental signaling pathways related to the onset and progression of CHDs.

In addition to wall shear stresses, cardiac pressures build during embryonic cardiac development, with rapid pressure growth after the development of cardiac valves. For example, systolic pressure drop is around 1 mmHg during the fully looped embryonic chick heart, and the pressure drop in the mature adult chicken heart is 120 mmHg. (Lin and Taber, 1994). Our models do not account for valves, which form later in cardiac development, but still predict similar values to the looped embryonic heart. Pressure produces stresses in the heart wall, which influence critical signaling cascades for cardiac development and remodeling (Qi et al., 2015; Throm Quinlan et al., 2011; Watson et al., 2012; Morgan and Black, 2014). Newtonian models misrepresent pressure loads compared to non-Newtonian models. Pressures remain within the same order of magnitude between Newtonian and non-Newtonian simulations, but non-Newtonian models are a better representation of blood viscosity *in vivo*. Increased, and possibly pathological, ballooning is induced by high pressure loads on chamber walls. Furthermore, recent work by Dietrich *et al.* demonstrated endothelial cell proliferation and cell migration induced by flow contributed to chamber growth and ballooning in the embryonic zebrafish heart (Dietrich et al., 2014). The inclusion of endocardial cushions, decreasing the luminal diameter where the tube bends, could elucidate pressure differences in pre-chamber sections of the looping heart. Our data suggest that differences in pressure values predicted by Newtonian and non-Newtonian models may induce inaccuracies in reported pressure loads during embryonic cardiac development and CHD development.

Differences in Newtonian and non-Newtonian wall shear stresses and pressures are likely resultant of differences in velocity profiles between Newtonian and non-Newtonian cases and decreased viscosities at higher shear rates in non-Newtonian cases. Experimental models utilizing flow imaging techniques often use a Newtonian viscosity approximation to scale calculated shear rates to shear stresses and to evaluate pressures (Peterson et al., 2012). Viscosities ranging from 0.001 Pa·s to 0.008 Pa·s have been used, resulting in a wide range of stresses reported (Wang et al., 2006). Circumferential strains induced by luminal pressure loads and wall shear stresses affect gene expression in both endothelial cells and cardiomyocytes (Chien et al., 1998). Using a non-Newtonian model to predict stresses and pressures is more representative of the behavior of embryonic blood during embryonic cardiac development. Stresses and pressures predicted by non-Newtonian models could improve *in vitro* tissue engineered models of embryonic cardiac development and could better evaluate mechanotransducive sensitive pathways in cardiac cells. Furthermore, inaccurate magnitudes of wall shear stress and pressures from Newtonian models could be closer to pathological values and could misevaluate flow conditions during CHD formation and progression.

Several limitations should be considered when interpreting these results. First, the geometries are idealized representations of looping morphologies and do not represent subject-specific embryonic anatomies. Second, the present models assume rigid, stationary walls and therefore do not account for fluid–structure interactions, tissue compliance, or peristaltic wall motion known to occur in the developing heart tube. Third, a power-law rheological model was used to represent shear-thinning behavior and does not capture all features of embryonic blood rheology. Fourth, flow boundary conditions relied on extrapolated developmental cardiac output estimates. Consequently, absolute hemodynamic quantities should be interpreted with caution. Nevertheless, because geometry-specific boundary conditions were held constant between Newtonian and non-Newtonian simulations, the relative differences reported here provide insight into the isolated influence of blood rheology during cardiac looping. Future work should incorporate image-derived geometries, compliant walls, fluid–structure interaction (Lee et al., 2013), and alternative constitutive models to further refine developmental hemodynamic predictions. Moreover, the present study did not include formal sensitivity analyses for hematocrit, flow amplitude, or rheological parameters. Instead, these quantities were held constant to isolate differences attributable solely to rheological model selection. Future studies should also systematically evaluate the interaction between hematocrit, developmental flow rates, geometry, and constitutive model selection.

We conclude that numerical simulations of blood flow through stages of cardiac looping using a Newtonian model of blood viscosity overpredict wall shear stresses and pressures compared to a non-Newtonian model of blood viscosity. Simulations predicted local maxima in wall shear stresses where atria, outflow tract, and cardiac cushions develop during later stages of heart looping. Additionally, non-Newtonian simulations more evenly distributed wall shear stresses, which could affect ventricular trabeculation. Predicted upstream pressures could modulate ventricular ballooning. Future experimental studies should examine the relationship between non-Newtonian flow conditions and downstream mechanobiological responses in cardiac cells. We suggest that non-Newtonian viscosity models be used alongside Newtonian approximations when evaluating embryonic hemodynamics, as they provide a more physiologically relevant representation of blood behavior during cardiac morphogenesis. We suggest that non-Newtonian viscosity models of embryonic blood be used in alongside Newtonian approximations when evaluating hemodynamics during embryonic cardiac development, and that non-Newtonian models can aid in our knowledge of flow patterns and stresses during physiological and pathological cardiac morphogenesis.

## Data Availability

The raw data supporting the conclusions of this article will be made available by the authors, without undue reservation.
