# Supplementary material for: Non-Newtonian blood rheology significantly alters hemodynamic predictions during cardiac looping: a computational study
Source: Front Bioeng Biotechnol. 2026 Sep 23;14:1892144. doi: 10.3389/fbioe.2026.1892144 (PMC13643127; doi:10.3389/fbioe.2026.1892144)
Supplement: Supplementary file 1 [file DataSheet1.pdf]

Supplemental Information for:

Non-Newtonian Blood Rheology Significantly Alters Hemodynamic Predictions During Cardiac Looping: A Computational Study

Matthew C. [1,2] , Erica C. Kemmerling [2,+], Lauren D, Black III [1,2,*,+]

[1] Department of Biomedical Engineering, Tufts University, Medford, MA, USA

[2] Department of Mechanical Engineering, Tufts University, Medford, MA, USA

[3]Cellular, Molecular and Developmental Biology Program, Graduate School of Biomedical Sciences, Tufts University School of Medicine, Boston, MA, USA

[+] Authors contributed equally to this work

[*] Corresponding author

Corresponding Author

Lauren D. Black III

Department of Biomedical Engineering, Tufts University

4 Colby Street

Medford, MA 02155

Phone: 617-627-4660; Fax: 617-627-3231 ; Email: lauren.black@tufts.edu

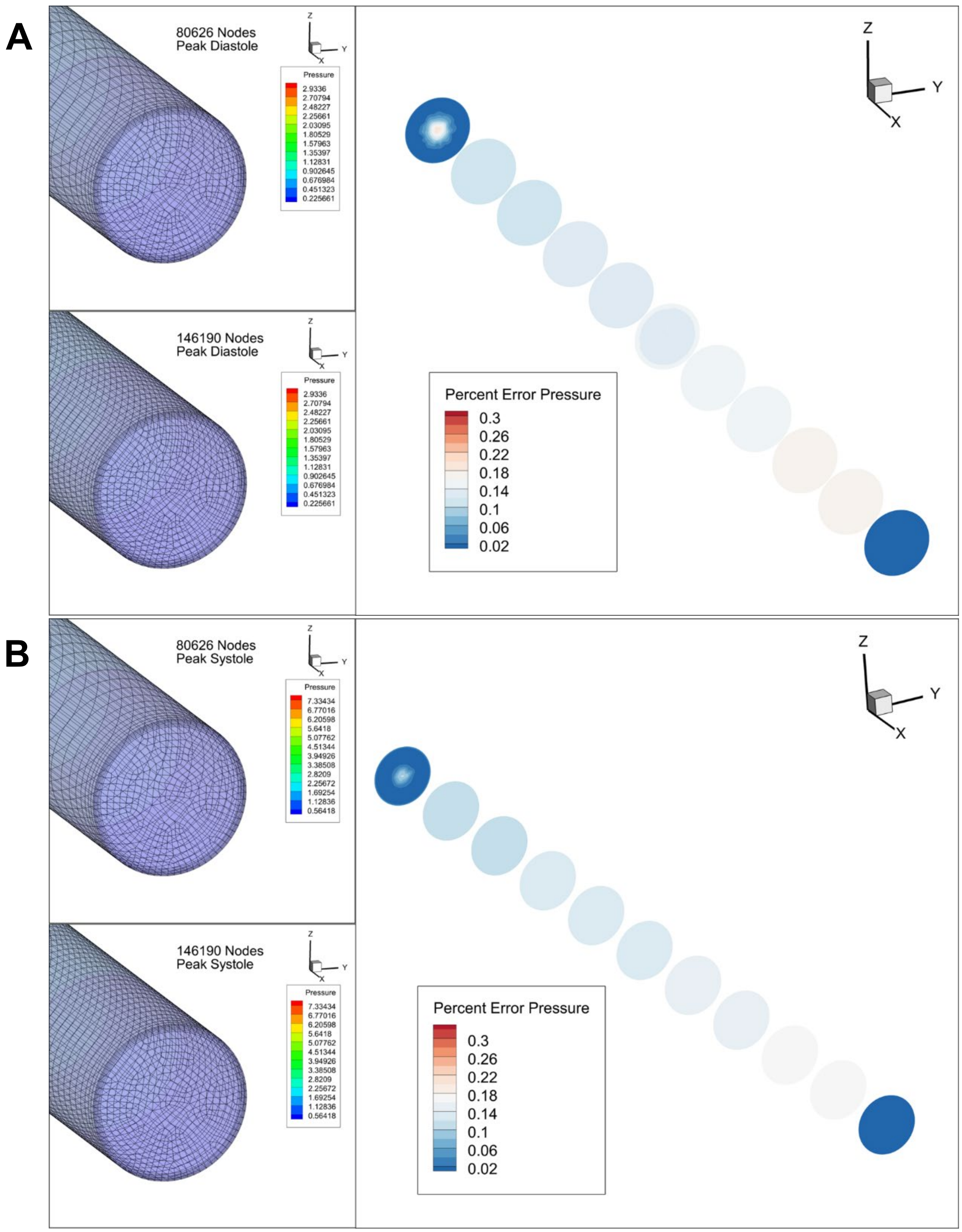

Supplemental Figure 1: Mesh convergence results for Stage 2 for both Peak Diastole (**A**) and peak systole (**B**) showing the error in pressures when moving from 80,626 nodes to 146,190 nodes.
